# A case of choroidal melanocytosis observed by multimodal imaging with laser speckle flowgraphy

**DOI:** 10.1186/s12886-023-02933-1

**Published:** 2023-04-26

**Authors:** Mizuho Mitamura, Satoru Kase, Kiriko Hirooka, Susumu Ishida

**Affiliations:** Department of Ophthalmology, Faculty of Medicine, Graduate School of Medicine, Hokkaido University, N-15, W-7, Kita-ku, Sapporo, 060-8638 Japan

**Keywords:** Choroidal melanocytosis, Laser speckle flowgraphy, Optical coherence tomography

## Abstract

**Background:**

Choroidal melanocytosis is characterized by congenital diffuse melanin pigmentation with extensive parenchymal infiltration of spindle cells in the choroid; however, little is known about the choroidal circulation and morphological changes. We herein report a case of choroidal melanocytosis observed by multimodal imaging with laser speckle flowgraphy (LSFG).

**Case Presentation:**

A 56-year-old woman was referred to our hospital because of serous retinal detachment (SRD) in her left eye. At the initial examination, her best-corrected visual acuity (BCVA) was 1.5 oculus dexter (OD) and 0.8 oculus sinister (OS). An irregular, flat, brownish lesion was noted around the macula OS. Optical coherence tomography showed a choroidal structure with marked hyporeflectivity and SRD where the retinal thickness was preserved. Indocyanine green angiography demonstrated fluorescence blockade throughout. Fundus autofluorescence revealed enlarged macular hypofluorescence, suggesting chronic retinal pigment epithelium damage associated with prolonged SRD. B-mode echography showed no choroidal elevation. Based on the clinical findings, the left eye was diagnosed with choroidal melanocytosis. Four years and 10 months after the initial visit, her BCVA was 0.5 and SRD remained. During the entire period of observation, the mean blur rate (MBR) (mean ± standard deviation) of choroidal blood flow velocity on LSFG was 10.15 ± 0.72 arbitrary units (AU) OD and 1.31 ± 0.06 AU OS.

**Conclusion:**

Choroidal melanocytosis presented with chronic minor circulatory disturbances due to melanocyte proliferation in the choroid, but the markedly low MBR values by LSFG were dissociated from her retinal thickness and visual function. The proliferation of melanocytes may be a cause of overestimating the cold-color signal of LSFG due to their pigmentation.

## Background

Choroidal melanocytosis is characterized by congenital diffuse or sector melanin pigmentation of the choroid without choroidal elevations [1, 2]. It results from incomplete migration of melanocytes from the neural crest [3]. On the other hand, melanocytoma is a benign neoplasm arising from the melanocytes, which is derived from neural crest cells [3]. Although melanocytosis and melanocytoma are believed to be the two extremes of the same congenital process of proliferation of pigmented cells, histological findings of both tumors are not identical [1]. Melanocytosis consists of spindle cells with widespread parenchymal invasion [1], while melanocytoma is a focal form which shows polygonal nevus cells [4, 5]. Understanding the differential diagnosis and pathophysiology with detailed evaluation of the choroid are of importance in the management of patients, since melanocytosis predisposes to uveal malignant melanoma [6, 7].

The fundus structure of choroidal melanocytosis is characterized by obscure choroidal vascular structures and a lack of overlying drusenoid degeneration, lipofuscin, and/or serous retinal detachment (SRD), indicating that the melanin pigmentation is isolated to Haller’s layer and without retinal pigment epithelium (RPE) disruption [8–11]. Hrynchak et al. reported that bilateral choroidal melanocytosis retained a normal retinal structure over the pigmented areas on optical coherence tomography (OCT) (Heidelberg OCT Spectralis), and fundus autofluorescence (FAF) revealed isoautoflourescence over the pigmented areas, suggesting no disruption of RPE or accumulation of lipofuscin [8]. In a comparison of affected and unaffected eyes in 15 patients with unilateral choroidal melanocytosis, enhanced depth imaging -OCT images showed the perivascular interstitial tissue enwrapping the vessels, and the stromal component of the choroid in the affected eye was 51% thicker than in the unaffected eye [12]. These results suggest that choroidal melanocytosis increased choroidal thickness as a result of increased tissue cellularity [12]. These choroidal structural changes in choroidal melanocytosis suggested that melanocyte proliferation might cause choroidal circulatory disturbance.

Laser speckle flowgraphy (LSFG) is a blood flow imaging method that uses laser scattering to noninvasively visualize the fundus circulation in two dimensions. We have used LSFG to view the fundus circulation in a variety of intraocular tumor-like lesions, including optic disc melanocytoma [13], choroidal macrovessel [14], sclerochoroidal calcification [15], juxtapapillary retinal capillary hemangioblastoma [16], choroidal lymphoma [17], and leukemic retinopathy [18]. Especially, optic disc melanocytoma showed a low signal on LSFG, in which the loss of vascular structures observed by OCT angiography (OCTA) was associated with visual field defects [13]. However, the details of choroidal circulation and morphological changes of choroidal melanocytosis are unknown.

We herein present a case of choroidal melanocytosis observed by multimodal imaging and analyze the choroidal circulation using LSFG.

## Case presentation

**Fig. 1:**
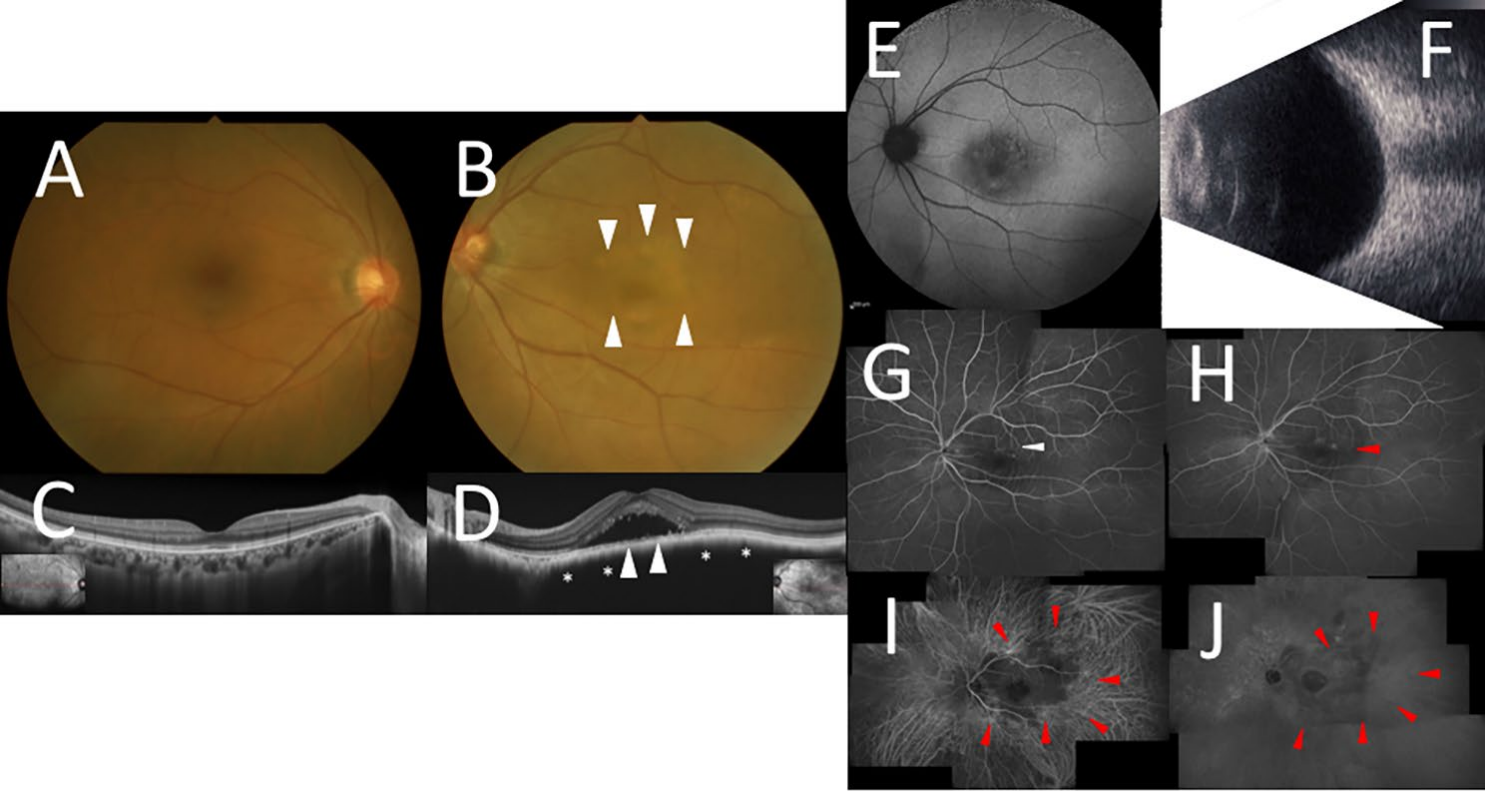
Initial findings on color fundus photography (CFP), swept-source optical coherence tomography (SS-OCT), fundus autofluorescence (FAF), B-mode echography, fluorescence angiography (FA), and indocyanine green angiography (ICGA) in the present case with choroidal melanocytosis. **A. B.** CFP showed no abnormality OD, while an irregular pale brown lesion was noted around the macula OS (white arrowheads) **C. D.** SS-OCT on horizontal scans through the fovea demonstrated no abnormalities of the retinal choroidal thickness or structure OD, while a choroidal structure with marked hyporeflectivity and SRD were noted OS. **E.** FAF indicated enlarged macular hypofluorescence OS. **F.** B-mode echography showed no obvious elevated lesions OS. **G. H.** FA revealed reduced choroidal perfusion in the macula in the early phase and scattered focal hyperfluorescent findings that intensified from early to late phase OS. **I. J.** ICGA demonstrated map-like fluorescence blockade throughout OS.

A 56-year-old woman had been followed up for two years before the initial visit with suspected age-related macular degeneration oculus sinister (OS) at a previous clinic. One year before the initial visit, her best-corrected visual acuity (BCVA) was 0.9 OS, and fluorescence angiography (FA) showed leakage of fluorescence at the macula, which indicated the possibility of central serous chorioretinopathy at the clinic. One month before the initial visit, SRD was observed with BCVA of 0.5 OS, and local photocoagulation was performed. However, due to residual SRD and worsening visual impairment OS, she was referred to our hospital. Her medical history was limited to the inguinal hernia surgery 20 years ago, and she had neither a smoking habit nor family history. At the initial examination, her BCVA was 1.5 oculus dexter (OD) and 0.8 OS, with normal intraocular pressure oculi uterque (OU). Slit-lamp microscopy did not detect any findings OU. Color fundus photography (CFP) showed no abnormality OD (Fig. 1A), while an irregular brownish lesion was noted around the macula OS (Fig. 1B, white arrowheads). Therefore, coloration of the posterior pole in the left eye was generally more yellowish-brown than the right eye. Although swept-source (SS)-OCT images (DRI OCT Triton; Topcon Inc., Tokyo, Japan) demonstrated no abnormalities of retino-choroidal structures OD (Fig. 1C), SS-OCT showed the markedly hyporeflective choroidal structure OS (Fig. 1D, asterisks) as well as SRD where the retinal thickness was preserved. Moreover, the lumen of the choriocapillaris was suggested to be compressed beneath the RPE layer OS (Fig. 1D, white arrowheads). FAF indicated enlarged macular hypoautofluorescence suggesting chronic RPE damage OS (Fig. 1E). There was no choroidal elevation on B-mode echography OU (Fig. 1F). FA revealed reduced choroidal perfusion in the macula in the early phase (Fig. 1G, white arrowhead) and scattered focal hyperfluorescent spots that intensified from early to late phase OS (Fig. 1H, red arrowhead). Indocyanine green angiography (ICGA) demonstrated serpiginous-like fluorescence blockade throughout OS (Fig. 1I J, red arrowheads). There was no obvious abnormal shadow on orbital magnetic resonance imaging. Based on the clinical findings, her left eye was diagnosed with choroidal melanocytosis together with SRD. Central serous chorioretinopathy was ruled out by the absence of choroidal thickening on SS-OCT, descending tract on FAF, and choroidal vascular hyperpermeability on ICGA. The absence of choroidal elevation observed by fundus examination and echography, and irregular melanin pigmentation of the choroid supported the diagnosis of choroidal melanocytosis rather than choroidal nevus. She was observed without any treatment. Fifty-eight months after the initial diagnosis, her BCVA was 0.5, CFP showed slightly darker macular pigmentation, and SRD persisted on SS-OCT.

The institutional review board of Hokkaido University waived the need for ethical assessment of this clinical study because of it being a single case report with a non-invasive study. This study adhered to the tenets of the Declaration of Helsinki.

**Fig. 2:**
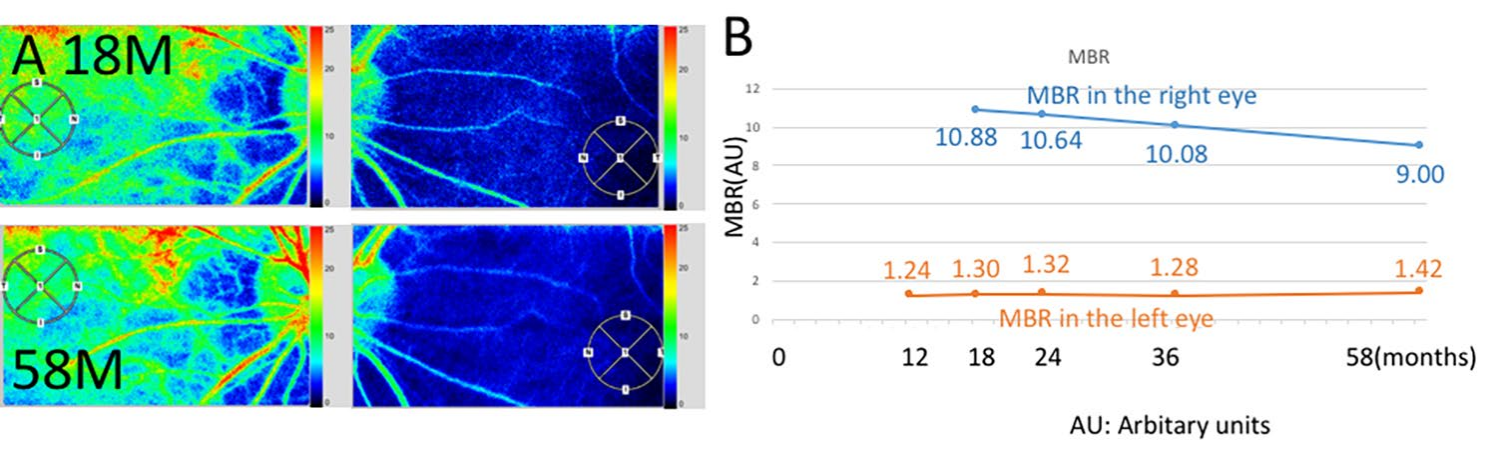
Laser speckle flowgraphy (LSFG) and mean blur rate (MBR) values during the course of the present patient with choroidal melanocytosis. **A.** LSFG at 18 and 58 months after the initial visit showed a marked cold-color blood flow signal of the left eye compared to with right eye. A circle of 750 μm in diameter to the fovea was defined as the region of interest on LSFG. **B.** The MBR values were consistently lower in the affected eye compared with the healthy eye over the entire course of the study

This study evaluated the alterations of choroidal blood flow of choroidal melanocytosis using LSFG. Relative blood flow values were obtained as the mean blur rate (MBR) after quantitative measurement of blood flow velocity by LSFG software (LSFG-NAVI, version 3.1.39.2, Softcare Ltd., Fukuoka, Japan) according to previous reports [18, 19]. The pupils of the patient were dilated with 0.4% tropicamide (Mydrin-M; Santen Pharmaceutical Co., Ltd., Osaka) before examination. Ophthalmic examinations were conducted after pupils of both eyes had completely lost their light reflex. The macula in the LSFG images was manually marked and vessels were automatically segmented using threshold values defined by the system software (LSFG Analyzer, version 3.0.47.0). The macular area was identified by an experienced examiner by comparing the initial FA images and FAF images. Since LSFG images also show retinal blood vessels, the macular area was determined by comparing their length and vascular runways with the FA images. A circle of about 750 μm in diameter to the fovea was defined as the region of interest on LSFG (Fig. 2A, small circles), based on FA findings. Four to five consecutive measurements were taken for each circle, and the mean values were used for analysis. All examinations were conducted by a single experienced operator. Ocular perfusion pressure (OPP) was calculated using the patient’s blood pressure and intraocular pressure, as previously described [19, 20].

The MBR values OD are shown in Fig. 2B as follows: 10.9, 10.6, 10.1, and 9.0 arbitrary units (AU) at 18, 24, 36, and 58 months after the initial visit, respectively. The MBR values OS are shown in Fig. 2B as follows: 1.24, 1.30, 1.32, 1.28, and 1.42 AU at 12, 18, 24, 36, and 58 months after the initial visit, respectively. The MBR (mean ± standard deviation) for 58 months was 10.15 ± 0.72 AU OD and 1.31 ± 0.06 AU OS. OPP was 82.2, 76.2, 82.9, 73.0, and 68.0 mmHg OD, and 83.2, 78.2, 82.9, 72.0, and 69.0 mmHg OS at 12, 18, 24, 36, and 58 months after the initial visit OS, respectively, revealing no significant changes in either eye.

## Discussion and conclusion

The present study demonstrated novel findings of choroidal melanocytosis via multimodal imaging techniques to better understand choroidal circulatory dynamics, especially with LSFG with long-term follow-up. To the best of our knowledge, this report is the first to show choroidal blood flow impairment in choroidal melanocytosis.

In this case, SS-OCT showed marked hyporeflective choroidal structures, which might correspond to histologically increased stromal tissue due to melanocyte proliferation. ICGA showed marked fluorescence block, where there was no choroidal elevation on B-mode echography. Based on the structural and angiographic findings, the patient was diagnosed with choroidal melanocytosis. A defect in both the myoid zone and ellipsoid junction was noted in one case [12], but there were no cases with SRD or RPE abnormalities according to the literature. In addition, FAF demonstrated enlarged macular hypoautofluorescence, and FA showed window defects suggesting chronic RPE damage, caused by a circulatory disturbance due to choroidal vascular compression, leading to prolonged SRD. On the other hand, although there was prolonged SRD, the retinal thickness and layer structure were preserved, indicating that the retinal circulatory disturbance was not severe enough to cause thinning of the retina. Regarding the localization of melanocytes, they were likely to diffusely infiltrate into not only the choroidal stroma but also surrounding choroidal vessels. Interestingly, this case was further complicated by compression of the choriocapillaris beneath the RPE layer on SS-OCT (Fig. 1D), indicating that melanocyte proliferation did not fully extend to the choriocapillaris. Taken together, the oxygen/nutrient supply to RPE was not completely disrupted due to compression of the choriocapillaris, and the blood flow impairment was not severe enough to cause atrophy of the inner and outer retina.

Oxygen supply to the inner retina is provided solely by the retinal circulation, whereas oxygen supply to the outer retina is provided primarily by diffusion from the choroidal circulation, with negligible supply from the retinal circulation [21]. Through autoregulatory mechanisms and vessel wall remodeling, the oxygen volume delivered to the outer retinal tissues can be controlled to some extent by the extraction of oxygen from the retinal circulation. In fact, it has been suggested that photoreceptor cells receive oxygen not only from the choroidal circulation but also from the retinal blood flow in the presence of retinal vascular lesions, as shown below: in a study of early-stage diabetic mice, when the choroidal oxygen supply of photoreceptors was reduced due to decreased choroidal circulation, the photoreceptors extracted more oxygen from the retinal circulation to compensate, indicating an increased oxygen supply to the outer retina [22]. Thus, it is possible that even if SRD was present for long in this case, the blood supply from the inner retinal layer preserved the laminar structure of all layers of the retina.

However, despite the preservation of the retinal layer structure, the marked cold-color signal of LSFG and extremely low MBR values in the Japanese patient with choroidal melanocytosis were unjustified. Hypothesized mechanisms for the unjustified cold-color signal of LSFG are discussed as follows.

LSFG utilizes a laser probe with a relatively long wavelength of 830 nm, which enables recording of choroidal blood flow, but the signal intensity generated by the choroid is largely dependent on the level of pigment content of RPE and/or choroidal melanocytes. Although there are no reports comparing LSFG results between healthy Caucasian and Asian subjects, LSFG confirms vascular patterns of choroidal origin as well as ICGA and may be used to evaluate choroidal hemodynamics in various choroidal diseases [23]. It is indisputable that Caucasian subjects exhibit lower levels of fundus pigmentation as compared with people of Asian descent [24]. Therefore, the marked cold-color signal of LSFG in this case did not directly manifest a significant reduction in choroidal blood flow, but was a combination of the effects of melanocyte pigmentation and a minor reduction in choroidal blood flow. The marked cold-color signal of LSFG was overestimated because there was a dissociation between the normality of the retinal layer structure and choroidal circulatory disturbance seemingly detected with LSFG.

Regarding the long-term course of choroidal melanocytosis, Augsberger et al. reported no enlargement of unilateral choroidal melanocytosis during the 6-month to 13-year observation period [9], and the two bilateral lesions in their report showed no evidence of growth during the 2- and 6-year observation periods [25]. In our case, OCT showed markedly low reflection that did not depict the choroidal blood vessel lumens, and ICGA showed fluorescence blockade throughout. However, anatomically, the vessel configurations are considered to exist there while the retinal thickness was preserved. These findings remained unchanged over the course of 4 years, indicating that the retinal functions including photoreceptors were preserved, although there was some degree of choroidal circulatory disturbance associated with melanocyte proliferation. During long-term follow-up, her BCVA remained at 0.5 for approximately 4 years without treatment. Taken together, choroidal melanocytosis is considered a slowly progressive choroidal circulatory disorder.

In conclusion, choroidal melanocytosis presented with choroidal circulatory disturbances due to melanocyte proliferation, but the marked cold-color signal of LSFG was dissociated from her retinal thickness and visual function. LSFG findings would be derived from the effects of melanocyte pigmentation as well as a minor reduction in choroidal blood flow. The marked proliferation of melanocytes in the central choroid may overestimate the cold-color signal of LSFG due to their pigmentation.

## Data Availability

N/A.

## Abbreviations

LSFG: laser speckle flowgraphy
SRD: serous retinal detachment
BCVA: best-corrected visual acuity
OD: oculus dexter
OS: oculus sinister
MBR: mean blur rate
AU: arbitrary units
OCT: Optical coherence tomography
FAF: Fundus autofluorescence
RPE: retinal pigment epithelium
OCTA: OCT angiography
FA: Fluorescence angiography
OU: oculi uterque
CFP: color fundus photography
SS: swept-source
ICGA: indocyanine green angiography
OPP: ocular perfusion pressure
